# 3D bioprinting technologies and biomaterial-based scaffolds for wound healing: Insights into decellularized tissue-derived bioinks

**DOI:** 10.1016/j.reth.2026.101147

**Published:** 2026-05-29

**Authors:** Ayda Khatibi, Mozafar Khazaei, Sepehr Zamani, Mostafa Al Jaf, Leila Rezakhani

**Affiliations:** a Department of Biological Sciences, Faculty of Basic Sciences, Institute of Higher Education of Nabi Akram, Tabriz, Iran; b Fertility and Infertility Research Center, Health Technology Institute, Kermanshah University of Medical Sciences, Kermanshah, Iran; c Department of Tissue Engineering, School of Medicine, Kermanshah University of Medical Sciences, Kermanshah, Iran; d Student Research Committee, School of Medicine, Shahroud University of Medical Sciences, Shahroud, Iran; e Student Research Committee, Kermanshah University of Medical Sciences, Kermanshah, Iran

**Keywords:** 3D printing, Extracellular matrix, Decellularization, Wound healing

## Abstract

The field of tissue engineering has witnessed significant advancements with the advent of 3D printing technologies, especially in wound healing. Innovative 3D-printed scaffolds incorporating decellularized biomaterials offer a promising approach to enhance the regenerative process. Decellularized biomaterials, derived from natural tissues, retain the extracellular matrix (ECM) components crucial for cell adhesion, migration, and tissue regeneration. These biomaterials are processed to remove cellular material, minimizing immune rejection and ensuring biocompatibility. When combined with the precision of 3D printing, these scaffolds can be tailored to match the specific needs of different wound types, promoting effective tissue integration and accelerated healing. This approach provides a platform for the creation of personalized, patient-specific treatments, addressing the limitations of traditional wound care strategies. Furthermore, the ability to print scaffolds with complex structures allows for the optimization of mechanical properties and porosity, facilitating nutrient exchange and cellular infiltration. This paper explores the potential of 3D-printed decellularized biomaterial scaffolds in revolutionizing wound healing, emphasizing their role in improving regenerative outcomes and reducing the risks of complications.

**Table:** 

Abbreviations	Definition
ECM	Extracellular Matrix
SLA	Stereolithography
FDM	Fused Deposition Modeling
Rep Rap	Replicating Rapid Prototyping
FDA	Food and Drug Administration
SLS	Selective Laser Sintering
GAG	Glycosaminoglycan
FGF	Fibroblast Growth Factor
EGF	Epidermal Growth Factor
VEGF	Vascular Endothelial Growth Factor
SFF	Solid Free-form Construction
PVA	Polyvinyl Alcohol
PB	Powder Bed
SFS	The Shape of the Shadow
WCE	Wireless Capsule Endoscopy
PAM	Pressure-assisted Microsyringe
HPMC	Hydroxypropyl Methylcellulose
CT	Computed Tomography
PLA	Polylactic Acid
PLGA	Polylactic-co-glycolic Acid
SDS	Sodium Dodecyl Sulfate
HHP	High Hydrostatic Pressure
WHO	World Health Organization
MSC	Mesenchymal Stem Cell
CAD	Computer-Aided Design Software
dECM	Decellularized Extracellular Matrix
ADR	Adverse Drug Reaction

## 1 Introduction

3D bioprinting is a cutting-edge technology that merges 3D printing techniques with biological materials to create tissue-like structures using "bio-inks," which are substances made up of living cells and biomaterials. This process enables the creation of tissues that mimic natural functions, with applications in regenerative medicine, drug testing, cancer research, and organ transplants [1,2]. Recent innovations in 3D printing and tissue engineering have opened up new possibilities for improving wound healing processes. One of the most promising developments is the use of 3D-printed scaffolds. These scaffolds serve as temporary structures that support tissue regeneration by mimicking the natural extracellular matrix (ECM) of the human body [3,4]. The combination of 3D printing technology with decellularized biomaterials, which retain the native properties of tissues while removing cellular content, provides a unique platform for creating customized, biocompatible, and functional wound healing solutions [5]. Despite advancements, challenges remain in creating complex tissues with integrated vascular systems and ensuring bioprinted organs. These innovative scaffolds not only promote the regeneration of skin and other tissues but also offer a higher level of precision and adaptability compared to traditional wound healing treatments [6]. By integrating advanced manufacturing techniques, researchers have been able to design scaffolds that cater to individual patient needs, providing a personalized approach to wound care. Moreover, the ability to fabricate these scaffolds with exact specifications allows for better integration with the patient's tissues, enhancing healing outcomes and reducing complications such as infections or scarring [7].

Current research is focused on addressing these challenges, with the potential to transform healthcare by offering personalized treatments and tackling organ shortages [8]. The field of wound healing has made significant advancements over the past few decades, but there is still a pressing need for more effective treatments, especially for chronic wounds and complex injuries [9]. 3D bioprinting has emerged as a transformative technology in wound healing, enabling the precise deposition of living cells, growth factors, and biomaterials to create customized scaffolds that promote tissue regeneration. This approach provides multiple benefits compared to traditional wound healing methods, such as the ability to customize treatments based on the specific needs of each patient [[10], [11], [12]].

In summary, 3D bioprinting represents a transformative approach in medical science, offering new avenues for tissue engineering, disease modeling, and regenerative therapies. Our research focuses on harnessing the potential of 3D-printed decellularized biomaterial scaffolds to address the gaps in current wound healing therapies. Despite advancements, current treatments still have limitations that do not fully address the needs of patients, especially those with chronic wounds that are difficult to heal. By exploring the distinct properties of decellularized biomaterials and harnessing the precision of 3D printing, we aim to develop more effective, personalized solutions that enhance healing times and minimize the risk of complications. Through our research, we hope to contribute to the development of a new era in wound healing, offering a more sustainable and personalized approach that will ultimately enhance the quality of life for those suffering from difficult-to-heal wounds. Ongoing research and technological progress are anticipated to address current challenges, clearing the path for wider clinical applications in the near future.

## 2 3D-printed scaffolds

The inception of 3D printing, or additive manufacturing (AM), dates back to the late 1970s, reaching a clinical milestone in the mid-1980s with Charles Hull's invention of stereolithography (SLA) [[13], [14], [15]]. This was followed by the development of key technologies such as Fused Deposition Modeling (FDM) and Binder Jetting, which provided the technical foundation for layer-by-layer material fabrication [16,17]. While initially used for industrial prototyping, these technologies rapidly transitioned into healthcare, moving from surgical planning and patient-specific anatomical models to the production of customized implants [18,19]. The integration of AM into the pharmaceutical and biomedical fields has since evolved from solid dosage forms to the fabrication of complex tissue scaffolds. This technological trajectory has paved the way for 'bioprinting,' where the focus shifted from inert polymers to bioactive materials, aiming to mimic the natural ECM for regenerative purposes [20].

### 2.1 Clinical significance of skin regeneration and the role of scaffolding

Human skin is a complex, multi-layered organ that serves as the primary barrier against environmental pathogens and physical trauma. Its structural and functional integrity is maintained by a sophisticated ECM rich in collagen, glycosaminoglycans (GAGs), and specialized cells such as keratinocytes and fibroblasts [21,22]. When this intricate balance is disrupted by chronic wounds or severe injuries, a highly regulated repair process involving growth factors (e.g., FGF, EGF, and VEGF) is triggered [23,24]. However, conventional treatments often fail to restore the full functional architecture of the skin. In the context of tissue engineering, 3D-printed scaffolds have become pivotal by providing the necessary spatial cues and mechanical support for cell infiltration and tissue remodeling [25]. Unlike generic scaffolds, advanced bio-fabricated constructs aim to replicate the skin's macro- and micro-environments to optimize nutrient delivery and cell-matrix interactions [26]. Therefore, developing bioinks that can accurately mimic the native skin ECM—such as decellularized tissue-based inks—is essential for achieving true regenerative outcomes in chronic wound management [27].

### 2.2 3D bioprinting technologies for bioink fabrication

The transition from traditional scaffold fabrication (e.g., solvent casting or electrospinning) to 3D bioprinting has enabled the creation of complex, patient-specific anatomical structures with precise internal microarchitecture [28,29]. Unlike industrial 3D printing, which often focuses on metals and ceramics, bioprinting for wound healing requires techniques that are compatible with delicate biomaterials and living cells [30,31]. Currently, several bioprinting modalities are utilized to process decellularized extracellular matrix (dECM) and other hydrogels, each offering distinct advantages and limitations. Among these, Extrusion-Based Bioprinting (EBB), which evolved from Fused Deposition Modeling (FDM) and Pressure-Assisted Microsyringe (PAM) technologies, stands as the most widely adopted method for dECM-based bioinks [[32], [33], [34], [35]]. This technique involves the continuous extrusion of a pre-gel through a micro-nozzle, providing the unique capability to process high-viscosity materials and high cell densities at ambient temperatures—a feature that is paramount for preserving the sensitive bioactive proteins and growth factors inherent in dECM [36].

In addition to extrusion methods, Stereolithography (SLA) represents a prominent light-based approach that employs UV or visible light to crosslink photosensitive bioinks in a layer-by-layer fashion. While SLA is recognized for its superior spatial resolution (down to <25 μm), its integration with dECM bioinks is often challenged by the requirement for chemical functionalization of the matrix, such as methacrylation, and the potential genotoxic effects associated with UV exposure [37,38].

Another approach involves Selective Laser Sintering (SLS), which constructs three-dimensional architectures by sintering powdered substrates with a high-energy laser. Although SLS-printed constructs exhibit robust structural integrity, the intense thermal energy required for the process frequently leads to the thermal degradation of the essential signaling molecules required for effective wound healing. As a result, the application of SLS in skin tissue engineering remains largely confined to the development of rigid, acellular frameworks rather than bioactive, cell-laden grafts [39,40].

**Fig. 1:**
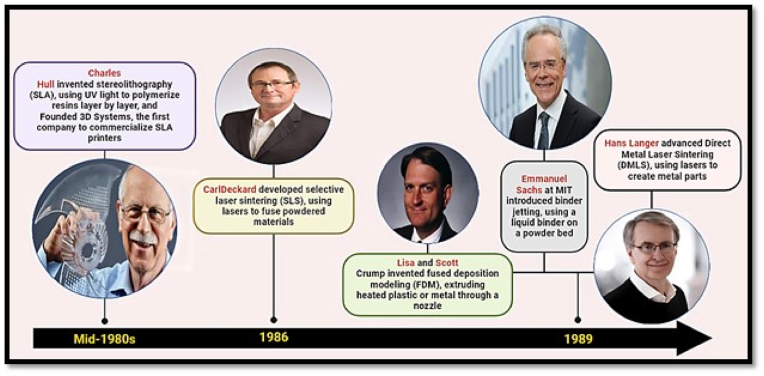
Timeline of key developments in 3D printing technology, from its initial patenting in the late 1970s to its subsequent advancements. Key innovations include stereolithography (SLA), selective laser sintering (SLS), and jet binder.

By integrating these Solid Free-form Fabrication (SFF) methods with patient-specific medical imaging (CT or MRI), researchers can now manufacture customized skin grafts that precisely match the anatomical contours of a wound, ensuring optimal integration and nutrient delivery [41,42] (see Fig. 1).

**Fig. 2:**
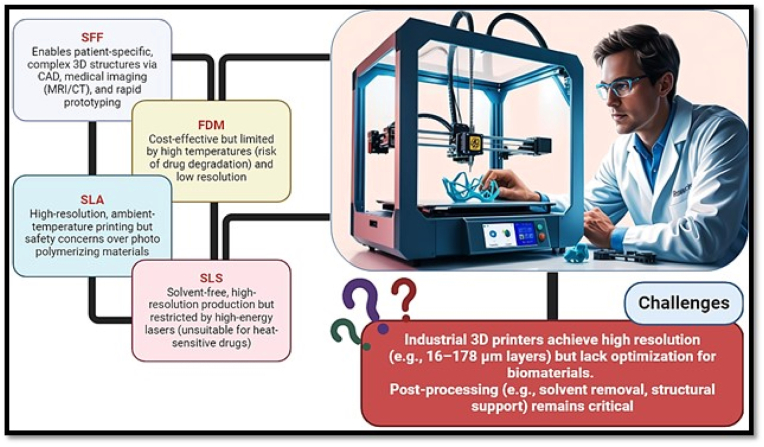
Overview of key 3D printing techniques, including stereolithography (SLA), selective laser sintering (SLS), fused deposition modeling (FDM), and solid freeform fabrication (SFF). Each method utilizes distinct processes and materials to create complex three-dimensional structures.

An ideal scaffold should ideally consider a comprehensive range of factors; however, challenges remain concerning biomaterial selection and the specificity of three-dimensional shapes. The biomaterials commonly employed include polymers (both synthetic and natural), ceramics, and metals. Each of these materials possesses distinct properties regarding composition, mechanical characteristics, processing techniques, chemical behaviors, interactions with cells, and regulatory approval by the FDA [43] (Fig. 2).

### 2.3 Advantages of 3D bioprinting in wound management

The integration of 3D bioprinting into wound care offers transformative advantages over traditional skin grafting and conventional scaffolding. By utilizing digital data from patient imaging techniques, such as laser scanning or CT, 3D bioprinting facilitates the development of custom-designed, personalized constructs tailored to the specific geometry of complex or irregular wound sites [44,45]. This precision ensures optimal contact between the printed graft and the host tissue, which is essential for effective integration and healing.

The primary benefit of using decellularized tissue-based bioinks (dECM) within this technology is the ability to create 'biologically active' implants that closely mimic the patient's native tissue [46]. Unlike synthetic scaffolds or titanium prostheses used in other surgical fields, dECM-based bioprinted constructs provide an inherent reservoir of tissue-specific signaling molecules [47,48]. This approach addresses a critical clinical need in reconstructive surgery by restoring not only the structural integrity of the skin but also its functional components, such as vascular networks and neural cues, without the need for invasive donor site procedures [49,50].

Furthermore, 3D bioprinting allows for the precise spatial distribution of multiple cell types and biomaterials in a single fabrication process. This capability is particularly beneficial for chronic and full-thickness wounds, where replicating the stratified architecture of the epidermis and dermis is vital for successful regeneration [51,52]. While challenges regarding clinical cost-effectiveness and regulatory approval remain, the potential of dECM-based bioprinting to eliminate the reliance on donor organs and improve surgical outcomes marks a significant advancement in personalized regenerative medicine [53,54].

## 3 Biomaterials in 3D-printed scaffolds

**Fig. 3:**
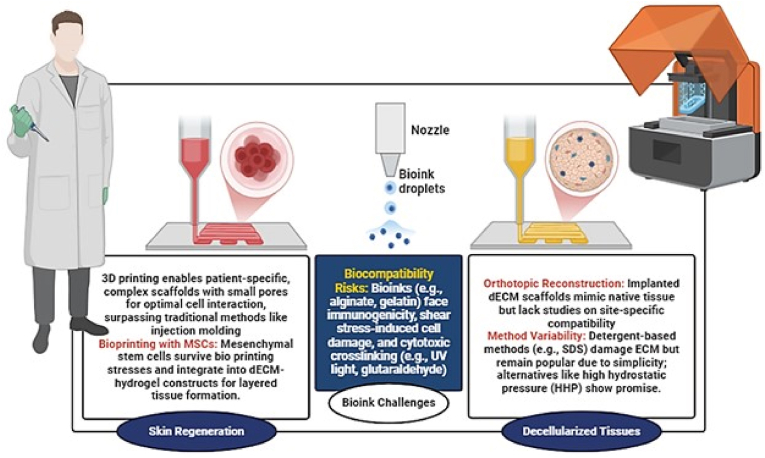
An overview of biomaterials and challenges in 3D bioprinting for tissue engineering. The left section highlights bioprinting with mesenchymal stem cells (MSCs) for skin regeneration, enabling patient-specific scaffolds with enhanced cell interaction. The right section focuses on decellularized extracellular matrix (dECM) scaffolds for orthotopic reconstruction, discussing method variability and compatibility concerns. The center emphasizes biocompatibility challenges, including bioink immunogenicity and stress-induced cell damage.

In tissue engineering and regenerative medicine, biomaterials used in 3D-printed scaffolds play a crucial role by providing structural support and delivering biological signals that promote cell attachment, growth, and development (Fig. 3). Tissue engineering for full-thickness skin regeneration requires materials that act as a temporary protective barrier for the wound bed. This approach is crucial for accelerating the healing process while restoring the skin's mechanical stability and elasticity [55,56]. In tissue engineering, three-dimensional printing plays a vital role, particularly in creating scaffolds. These scaffolds feature small pore sizes and large surface areas, which are essential for promoting optimal cellular interactions and supporting the formation of new functional tissues. Polylactic acid (PLA) represents significant progress in the field of biomaterials, due to its significant benefits, including biocompatibility and exceptional processing [57]. Current advances in 3D printing technology, particularly in the production of implantable biomedical devices, face significant limitations due to the limitations of available printed materials. Consequently, in numerous instances, it becomes necessary to employ alternative material processing techniques to accommodate materials that are not amenable to conventional printing methods [58]. When materials are suitable for printing, 3D printing provides considerable advantages in producing unique, customized complex devices that would be cost-prohibitive using traditional manufacturing methods, like injection molding [59]. Dermatological conditions are usually treated with a variety of topical therapies, such as antihistamines, antibiotics, laser treatments, localized vitamins, and corticosteroids [60]. Conventional wound healing techniques, such as skin allografts, amnion, and xenografts, present various challenges. These include issues with tissue antigenicity from donors, a higher risk of infection, and the absence of a basement membrane. As a result, skin tissue engineering has emerged as a progressively viable alternative, demonstrating encouraging outcomes in the domain of wound management [61]. Gasparotto et al. [62] developed 3D-printed scaffolds based on PLA and graphene/PLA and tested them with different cell types to assess how scaffolds influence cell behavior. The results demonstrated that the synthesized scaffolding promotes cell coordination and differentiation, enabling the integration of myoblasts into multi-core myotubes. Carabay et al. [63] evaluated a biocompatibility and toxicity test on human skin fibroblasts using 3D printed PLA scaffolding. Cell proliferation remained elevated even on the 18th day, demonstrating that PLA scaffolds maintain biocompatibility over an extended period. A range of natural and synthetic polymers have been utilized in bioengineered skin grafts for wound healing. For instance, a two-layer, three-dimensional scaffold was created using poly (lactic-co-glycolic acid) (PLGA) and alginate materials, highlighting their potential for wound healing applications. In vitro tests showed successful cell adhesion and proliferation on the tissue samples, while the PLGA layer effectively prevented bacterial invasion. Additionally, an in vivo wound healing study using a rat model revealed that the scaffold reduced wound size by 20.8% within just four days, with full wound closure achieved within twelve days [64].

### 3.1 Challenges of biological ink (bioink) selection

The selection of an appropriate bioink represents one of the most critical determinants of successful 3D bioprinting for regenerative applications. Independent of cell source, candidate biomaterials must demonstrate robust biocompatibility, minimal immunogenicity, and negligible cytotoxicity prior to clinical translation [65]. Comprehensive preclinical evaluation is therefore essential before advancing toward human application. Many commonly used bioink components are derived from non-human sources, including alginate (extracted from brown seaweed) and gelatin (frequently porcine-derived). Although these materials offer favorable printability and gelation characteristics, their xenogeneic origin may introduce risks of immune activation, inflammation, or pathogen transmission. Such concerns highlight the importance of purification strategies and rigorous biological assessment [66]. Biodegradability constitutes another fundamental requirement for regenerative bioinks. Ideally, the material should provide temporary structural support while progressively degrading in synchrony with new tissue formation [67]. However, the degradation process may generate by-products that enter systemic circulation, necessitating careful investigation of their metabolic clearance and potential toxicity, particularly with respect to hepatic and renal function [68]. Mechanical forces generated during extrusion-based bioprinting present an additional challenge. Shear stress within the printing nozzle can influence cell viability and activate mechanotransduction pathways, potentially altering stem cell differentiation patterns or cellular phenotype [69]. Optimization of viscosity, nozzle diameter, and extrusion pressure is therefore critical to balance print fidelity with cellular integrity.

Furthermore, many hydrogel-based bioinks require post-printing crosslinking to stabilize their three-dimensional architecture. Crosslinking methods may involve chemical agents, thermal treatment, enzymatic reactions, or photopolymerization. While effective in enhancing structural stability, these approaches may introduce cytotoxic effects or induce DNA damage. In particular, ultraviolet (UV)-mediated photopolymerization can generate reactive oxygen species and free radicals that exert genotoxic effects, even when immediate cellular damage is not apparent [70,71]. Consequently, the development of milder crosslinking strategies and visible-light or enzymatic systems has gained increasing attention in recent years.

Collectively, the selection of bioinks requires a delicate balance among printability, mechanical integrity, degradation behavior, and biological safety, underscoring the multidisciplinary nature of biofabrication design [70].

While these challenges persist for most hydrogel-based systems, decellularized extracellular matrix (dECM) bioinks offer a unique solution by inherently providing the complex biochemical signaling and architecture that synthetic or single-component natural polymers lack, thereby bypassing many of the bioactivity limitations discussed above.

### 3.2 Decellularized tissues (bioink)

dECM has emerged as a highly promising bioink platform in 3D bioprinting due to its ability to recapitulate the native tissue microenvironment. Unlike conventional hydrogel systems, dECM bioinks preserve tissue-specific structural proteins, including collagen types I and III, elastin, laminin, fibronectin, and glycosaminoglycans (GAGs), which actively regulate cell adhesion, migration, proliferation, and lineage commitment. These retained biochemical cues enhance regenerative outcomes, particularly in cutaneous wound healing applications [72,73].

For biofabrication purposes, decellularized tissues are typically lyophilized, milled into powder form, and enzymatically digested using pepsin under acidic conditions to generate a solubilized pre-gel solution. Following neutralization, neutralization to pH 7.4 and temperature adjustment, the dECM solution undergoes thermally induced fibrillogenesis at physiological temperatures (37 °C), forming a stable hydrogel suitable for extrusion-based bioprinting. This temperature-responsive gelation mechanism enables controlled deposition while preserving cell viability.

From a rheological standpoint, dECM bioinks exhibit shear-thinning behavior, a critical characteristic for extrusion bioprinting. Under shear stress within the printing nozzle, viscosity decreases, facilitating smooth extrusion; upon deposition, viscosity recovery supports structural fidelity. However, pure dECM hydrogels often demonstrate limited mechanical stability, necessitating reinforcement strategies such as hybridization with GelMA or structural support via polycaprolactone (PCL) frameworks [74].

Beyond structural support, dECM bioinks exert significant immunomodulatory effects during wound healing. Studies have demonstrated that decellularized matrices promote macrophage polarization toward an M2 regenerative phenotype, thereby reducing chronic inflammation and accelerating tissue remodeling. This immunoregulatory capacity is particularly advantageous in chronic and diabetic wounds, where persistent inflammation impairs healing progression [75].

Additionally, optimized decellularization protocols may preserve matrix-bound growth factors such as VEGF, FGF, and TGF-β. These residual bioactive molecules enhance neovascularization, granulation tissue formation, and re-epithelialization in full-thickness wound models, facilitating improved integration of bioprinted constructs with host tissue.

Importantly, the choice of decellularization method significantly influences the biochemical integrity and mechanical performance of the resulting bioink. Chemical, enzymatic, and physical approaches differentially affect collagen architecture, GAG content, and growth factor retention. Therefore, careful optimization of decellularization protocols is essential to balance efficient cellular removal with preservation of ECM bioactivity, ultimately determining the regenerative potential of dECM-based bioinks [[76], [77], [78]].

## 4 Applications in wound healing

In human health, wounds have always been a serious problem, especially due to complications from comorbidities such as diabetes and infection. According to the World Health Organization (WHO), about 58 million people have been exposed to fatal injuries, and the annual death rate is 5 million [79]. The remaining several million individuals require appropriate treatment and care. Several factors affect the wound healing process, the most important of which are the patient's acute or chronic wound, and the patient's physical condition, which is not ineffective in the healing process [80]. Wound healing is a complex process that generates many biological responses when a wound is formed. In this complex process, at the first stage, various immune cells, such as neutrophils, monocytes, macrophages, and lymphocytes, are combined with non-immune cells, such as endothelial cells, fibroblasts, and keratinocytes. In addition to the above, solution intermediates, including cytokines, growth factors, and ECM components, play an important role in the healing process [81,82]. The ability to print tissue-like structures by precisely delivering living cells along with the right materials, in a structured and organized way, at specific locations, in adequate quantities, and within the optimal environment, is crucial for a variety of emerging technologies [83]. Biofabrication technologies, such as bioprinting, have emerged as tissue engineering approaches for building organs and smaller organoids or tissue constructs [84,85]. Tissue repair strategies today primarily focus on tissue engineering [86], which involves creating functionalized materials that replicate native tissues. In this context, 3D bioprinting has emerged as a promising technique, enabling the simultaneous deposition of living cells within biocompatible biomaterials [87]. This approach allows for the creation of complex, precisely defined structures with custom dimensions using a layer-by-layer bio-fabrication process [88,89]. A key advancement in this field is the use of mesenchymal stem cells (MSCs), which are durable enough to withstand the shear stress and pressure during the bioprinting process [90]. MSCs can be sourced from various tissues, including bone marrow, adipose tissue, synovium, periosteum, and muscle. By combining MSCs with decellularized extracellular matrix (dECM) in bioinks, researchers can produce 3D tissues through a deposition process that incorporates custom-designed layers [91,92]. The bioinks typically used for tissue substitutes are hydrogels, which provide a balanced combination of biochemical and physical properties, supporting homogeneous cell encapsulation while maintaining structural integrity [[93], [94], [95], [96], [97]]. This makes hydrogels highly promising for applications in tissue engineering and regenerative medicine [83,98,99]. 3D printing, also known as additive manufacturing, involves the layer-by-layer deposition of materials to gradually build a solid model. The technology uses computer-aided design software (CAD) that passes the necessary instructions to a 3D printer. The printer then converts the digital model into two-dimensional (2D) cuts, enabling the production of solid layers that eventually form the object in question [100]. A key challenge in extrusion-based bio-fabrication methods is the integration between the deposition and fabrication hardware and the various types of biomaterials, or bio-inks, being used for deposition [101]. Standard hydrogels present design challenges because they are either printed as fluid solutions, which limits their mechanical properties, or printed as solid hydrogels, which break apart during the extrusion process. Other laboratories have previously explored options to solve these problems, including cell aggregate printing into hydrogel substrates [102,103], cell and hydrogel rod extrusion from microcapillary-based cartridges [[104], [105], [106]], dynamically crosslinking extrudable hyaluronic acid (HA)-gold nanoparticle hydrogels [107], temporal control of hydrogel stiffness using photopolymerizable methacrylated HA and gelatin [108], fast polymerizing UV-activated thiol-ene crosslinking [109], fibrinogen-thrombin-based crosslinking [110,111], and ionic exchange facilitated alginate-collagen gels [112]. These examples highlight the potential for successfully bioprinting materials, yet concerns remain regarding the outcomes of these studies, particularly the use of harsh cross-linking agents such as glutaraldehyde [113]. In addition to the chemical, mechanical, and temporal properties that enable these materials to be bioprinted, successfully creating viable and functional 3D tissue constructs requires considering additional factors that influence long-term cellular viability and function. Typically, cells stay fixed in their initially deposited positions throughout the culture period, as they cannot adhere to or degrade the surrounding alginate gel matrix [114]. The limited interaction between cells within the gel can be attributed to the non-interactive nature of alginate. As a result, despite some successful reports of bioprinted cell-printed structures, the primary concerns remain minimal cell-material interactions and suboptimal tissue formation [115]. These materials fall short of mimicking the complexity of natural extracellular matrices (ECMs) and, therefore, cannot recreate the cell-cell connections and 3D cellular organization characteristic of living tissues. As a result, cells within these hydrogels are unable to display the intrinsic morphologies and functions found in vivo. Ideally, cells should be provided with a microenvironment that closely resembles their native tissue. Decellularized extracellular matrix (dECM) is the most suitable option for this, as no natural or synthetic material can fully replicate all the features of a natural ECM [116].

Formed through dynamic and reciprocal interactions between the resident cells and their microenvironment [116]. Recent studies on cells and ECM extracted from tissues and organs emphasize the importance of tissue specificity in maintaining specific cell functions and phenotypes [[116], [117], [118], [119], [120]]. The dECM materials are harvested and typically processed as two-dimensional (2D) scaffolds from various tissues, including skin [121], and small intestinal submucosa [122], where, at the initial stages, the infiltrating or seeded cell populations depend on diffusion of oxygen and nutrients for their survival until a supporting vascular network develops. However, printing tissue analog structures requires a fabrication approach to devise a highly open porous 3D structure to allow the flow of nutrients [123,124]. Organ transplantation in an orthotopic position is regarded as the definitive treatment for end-stage organ failure [118,119,125]. The demand for transplantable organs exceeds the availability of donor organs, creating a significant need for the development of tissue and organs to address this gap. In response, we have developed a bioprinting method that utilizes novel dECM bioinks to print cell-laden structures, providing an optimized microenvironment that supports the growth of 3D tissue with reconstituted cellular morphologies and functions. Our bioprinting process demonstrates versatility and functionality across different types of dECM bioinks, including those derived from adipose, cartilage, and heart tissues. These cell-printed constructs show promising results in higher-order assembly, with organized spatial patterns and tissue-specific gene expression. A key advantage of this approach is the use of tissue-specific ECM, which offers essential cues for cell engraftment, survival, and long-term functionality. Despite the development of more than 3000 products aimed at wound treatment, this issue continues to pose a significant burden at both the societal and individual levels [126] (Table 1).

**Table 1:** Bioink based on decellularized tissues in skin regeneration.

Natural biomaterials-based bio-inks					
Biomaterial	Bioprinting method	Cell type	Target tissue	Cellular response	Ref.
Three different concentrations of ECM solution (0, 1.5, and 3.5%) were mixed with an alginate gelatin solution (6%–6%) to create ECM-Alg-Gel bio-ink.	3DPL N2 Plus Bioprinter (3DPL, Iran) and CAD/CAM software were used to evaluate the printability.	Human placenta	Skin Tissue	The macroscopic, microscopic, and molecular examinations of wound healing were evaluated in vivo and compared with those wounds implanted with Alg/Gel and injuries with no treatment (control).	[127]
3D bioprinted dECM/Gel/QCS (dGQ) hydrogel scaffold loaded with a novel antibacterial agent	An extrusion bioprinting technology with a bio ink composed of decellularized extracellular matrix (dECM), gelatin (Gel) and quaternized chitosan (QCS), and assembled with poly(ionic liquid)s (PILs) bonded by hydrogen bonds.	Decellularized ECM	Skin Tissue	These results revealed that GTMAC was successfully conjugated to the chitosan backbone.	[128]
A nanocellulose/alginate-based bioink was utilized.	Novel Passive Mixing Unit Technique	DsECM	Cartilage and Skin Tissue	Cell distribution appeared homogenous in both cases. Cells begin spreading by day 14, are highly spread by day 28, and indicate good viability.	[129]
Fibrinogen Hydrogel and Fibrinogen + dsECM Hydrogel	The integrated tissue organ printer (ITOP) system	Decellularized ECM	Skin Tissue	The findings demonstrate an increase in the mechanical strength of fibrinogen hydrogel supplemented with dsECM.	[130]
Collagen and Elastin	The 3D cell-laden structures composed of PSP-inks were produced using a custom-made 3D bioprinting system at the Korea Institute of Machinery and Materials (Daedeok-gu, Korea).	Decellularization of the porcine dermis	Skin Tissue	The nuclei of cells in decellularized skin were not stained compared to those in cells from normal skin, whereas the ECM of the tissue was maintained.	[131]
Fibrinogen and Collagen	To print a skin construct that exactly matches a patient's wound, the current prototype incorporates a real-time 3D laser scanner, ZScanner™ Z700 scanner (3DSystems, Rock Hill, SC).	The epithelium of the mouse	Skin Tissue	Treatment with autologous fibroblasts and keratinocytes, delivered directly to specific locations of the wound based on wound size and topology, resulted in the acceleration of wound healing and the formation of normal skin in situ.	[132]
A soft hydrogel is produced by neutralization with NaOH and incubation at 37 °C.	Gelatin Slurry Support Bath Printing	Skin-derived dECM (Fresh porcine abdominal skin)	Skin Tissue	Porcine skin-derived dECM was mainly comprised of dermal collagens and had retained additional matrisome molecules.	[133]

### 4.1 Challenges of cell source selection

In healthcare, the choice of cell sources and bioink materials can spark significant debate. For instance, cells used in creating simple tissue constructs, like heart valves, may originate from either animal or human sources, similar to the porcine valves currently used in clinical procedures. While animal-derived cells can aid in scaling up tissue production for surgical applications, this approach introduces the risk of using allogenic materials, which could potentially lead to disease transmission between species [134]. Human-derived sources demonstrate enhanced biocompatibility and the potential for personalization; however, their application is likely to encounter stricter regulations, prolonged production timelines, and increased costs. While the ethical concerns associated with donor-related practices may be alleviated through the utilization of autologous cell sources, challenges regarding the accessibility of specific cell types and the implications of existing genetic conditions may complicate the ethical and regulatory considerations surrounding autologous cell procurement [135]. Furthermore, the current limitations in human trials associated with the successful clinical translation of tissue-engineered constructs contribute to an inherent unpredictability concerning the behavior of autologous cells. The biological components of these implants lead to increased unpredictability in their integration and interactions with host organisms, in contrast to the more established devices such as stents, pacemakers, and artificial joints [136]. Differences in patient genotypes can impact several biological processes, such as cell migration, post-printing phenotypes, oncogenic potential, especially in immortalized cell populations, and abnormal differentiation. A notable example of such dysregulation is seen in adipose-derived stem cells, which can produce ectopic bone [137]. The formation of teratomas and the potential recurrence or worsening of malignancy associated with stem cell use pose major scientific challenges. A notable example is a first-in-human trial in Japan involving induced pluripotent stem cells, which was halted due to the development of genomic mutations [138,139].

### 4.2 Preparation of dECM bio-ink

In tissue engineering, combining biomaterials with cell-degenerated tissues creates bioinks by providing a 3D scaffold-like structure, derived from the ECM of the degenerated tissue, which can be used in 3D bioprinting to "print" new tissue constructs that closely mimic the natural tissue environment, allowing cells to adhere, proliferate, and differentiate into functional tissue to repair wounds; essentially, the biomaterial acts as a support system while the cell-derived ECM components provide crucial biochemical signals for cell behavior and tissue regeneration [140].

The diverse selection of biomaterials available, coupled with the ability to customize bioinks, is crucial for creating more realistic models that can be integrated into an in vivo environment for tissue regeneration [141]. Bioinks can be tailored with specific growth factors or signaling molecules to accelerate and enhance tissue regeneration in vivo. For instance, Lee et al. showed that embedding growth factors directly into bio-printed scaffolds improved their release and delivery, thanks to the 3D cell patterning within the scaffold [142]. Finally, 3D bioprinting allows for the creation of tissue components using the patient's own undifferentiated stem cells, sourced from their bone or fat marrow. Since these cells are immunotolerant, they minimize the risk of rejection during in vivo implantation, effectively preventing the graft-versus-host reaction. This represents a key advantage of 3D bioprinting [136,143]. Overall, employing 3D bioprinting for tissue regeneration will enhance the accuracy of recreating native morphology, anatomy, porosity, and other essential characteristics of the regenerated tissue.

The process of printing cell-laden constructs using dECM bioink involves several steps. The first step is the decellularization of the ECM material, with the primary goal being to remove as much cellular material as possible while minimizing the loss and damage to the ECM [144]. Therefore, it can be concluded that we successfully decellularized all the tissue samples. However, xenogeneic ECM derived from pigs and other animals may contain the Galα1,3Galβ1,4GlcNAc-R (Gal) epitope, a key contributor to the hyperacute rejection of pig organ transplants in primates. Despite the presence of the Gal epitope in porcine-derived ECM scaffolds, studies have shown that while it may increase serum antibody levels, no adverse reactions have been reported during tissue remodeling [145].

## 5 Challenges and future perspectives

3D printing technology has special potential in the design, application, and manufacture of various pharmaceutical products. Traditional methods are indeed affordable, but they often require a large workforce and waste a lot of time, especially if large-scale production is considered. In traditional manufacturing, making drugs in high doses to meet individual patient problems and needs poses significant challenges and problems. But today, 3D printing has undeniable potential in the healthcare and medical debate on the path to medical personalization. Additionally, the implementation of on-demand manufacturing within clinical settings can facilitate the delivery of optimal medical care [146].

## 6 What are the anticipated developments in 3D bioprinting in the next 20 years?

**Fig. 4:**
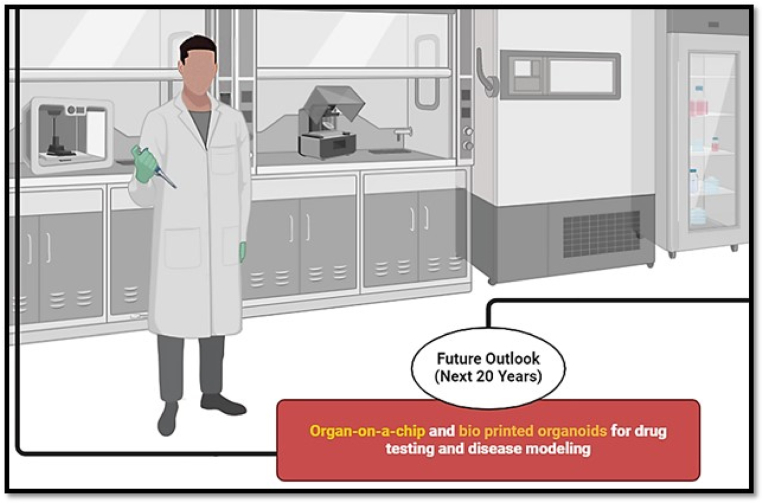
The future of 3D printing, highlighting advancements in bioprinting, sustainable materials, and AI-driven design. Emerging technologies aim to enhance precision, scalability, and applications across healthcare, and manufacturing.

Bioprinting, as a fabrication technology, combines software, hardware, and wetware processes to enable both high-throughput and precise placement of cells, biomolecules, and biomaterials in a spatially controlled manner [147] (Fig. 4). This multidisciplinary approach enhances the capability to construct complex biological structures with accuracy [148]. The unique characteristics of bioprinting make it an ideal technology for replicating native living organoids, tissues, and organs, effectively creating a "living environment" for applications in both translational medicine and research [149,150]. This technology builds on the core principles of tissue engineering, where tissue architecture is reconstructed through the careful selection of cell types, scaffolds, and growth factor combinations. Additionally, it is enhanced by the ability to customize, automate, and replicate the final tissue-engineered product [151]. The prevailing approach to medical treatment currently adheres to a “one size fits all” paradigm, wherein the majority of patients are administered the same medications at identical dosages and schedules as their counterparts [152]. Emerging evidence suggests that the paradigm of a “one size fits all” approach is insufficient across various therapeutic interventions [153]. The response may be either exaggerated, potentially associated with adverse drug reactions (ADRs), or insufficient, resulting in minimal or no pharmacological effects. Both scenarios can lead to additional complications for the patient [154]. The main purpose of skin tissue engineering is to facilitate the regeneration of the natural structure of the skin, thereby restoring the integrity of the structure and functional capabilities of the natural human skin. This approach is done by increasing the oxygen supply to the wound environment, normalizing the moisture level in the wound, and protecting against possible infections [155]. Despite the extensive range of commercially available skin substitutes, very few of them are regarded as ideal equivalents necessary for effective wound healing [24].

## 7 Ethical challenges

The design of clinical trials presents significant challenges, as it would be unethical to conduct trials on healthy volunteers for tissue-engineered organ transplantation. Furthermore, the utilization of patient-specific cell populations necessitates that patients serve as their controls, thereby introducing substantial heterogeneity in the assessment of treatment efficacy [135]. This situation may pose significant challenges in the interpretation of favorable outcomes observed in clinical trial participants. It is crucial to determine the extent to which observed effects can be attributed to the patient's inherent responses to the treatment, rather than the printed product itself. A structured and thorough framework for assessing the impact of printed interventions must be established before any clinical trials in this area are conducted. To date, clinical trials involving tissue-engineered constructs have primarily been carried out on patients with terminal illnesses. In these cases, such "last resort" treatments are often considered "more ethical," despite the uncertainties and potential complications they may introduce. One example is the use of skeletonized trachea from cadaveric sources, which are then seeded with patient-derived mesenchymal stem cells for use in surgical procedures [156]. In these cases, the key factor in obtaining ethical approval is the representation of the patient's clinical urgency. By framing the trial of a tissue-engineered trachea as a last-resort option and a final chance for lifesaving intervention, the process of securing ethical approval for its use in patients was made easier [45,157]. This method proved effective in advancing translational bioengineering; however, it represents a shortcut that introduces substantial limitations. The advancements in this field carry the risk of fostering uncontrolled and unethical practices. This concern is exemplified by the Macchiarini scandal, which involved the misleading representation of outcomes related to synthetic trachea implantation [158]. The lack of sufficient preclinical evidence in this case highlights the crucial need to establish a solid foundation of scientific and clinical validity before implementing clinical practices [46]. Designing clinical trials presents several challenges, notably the inability of patients to withdraw from the study following implantation. Additionally, obtaining informed consent for trial participation is complicated by the uncertainty surrounding the potential complications. One perceived advantage of three-dimensional structures is the inherent degree of reversibility associated with their removal should complications arise. In contrast, injectable stem cell and gene-based therapies may present significant challenges, potentially rendering them irreversible [135].

## 8 Conclusion

3D printing and bioprinting have the potential to be among the most transformative technological disruptions in healthcare and research this century. The integration of human cells and biocompatible materials into 3D printing practices is set to revolutionize surgical interventions, opening the door to the creation of living tissues and organs through advanced printing techniques. This could pave the way for custom-made body parts, reduce the need for organ transplants, and even eliminate the use of animals in pharmaceutical development and testing. As a result, patients could have access to personalized treatments throughout their healthcare journey. The global applications of bioprinting technology are already impressive, with successful innovations such as vascular systems, composite tissues, organoids, and complex cellular and tissue models. These breakthroughs are driving progress in drug development, cosmetic testing, and experimental research, highlighting the immense potential of bioprinting across various fields. The bioprinting market has seen substantial growth, with specialized companies now focusing on tissue production and manufacturing. Products like desktop 3D bioprinters, bioinks, and scaffolds (both with and without growth factors) are becoming more accessible, contributing to market valuations in the billions of dollars. However, this diversification of technology brings with it a challenge: the lack of a comprehensive "end-to-end" perspective, which makes it difficult for any single agency to fully grasp the technology's potential. This gap is a significant barrier to realizing the full scope of what 3D bioprinting can achieve. The successful translation of 3D printed constructs into clinical practice requires overcoming various hurdles. To optimize this pathway, a coordinated effort between scientists, engineers, and clinicians is necessary, all supported by an effective supply chain infrastructure. It is no longer enough for each group to work in isolation; collaborative approaches are essential to bring this transformative technology into the healthcare sector. For surgeons, fully harnessing the power of 3D printing in surgery requires both expertise in the field and a deep awareness of ongoing advancements in the technology. Identifying the right applications for 3D printing and integrating them into standard surgical practices will be key. As 3D printing and bioprinting continue to advance, their impact on the future of surgery is expected to be nothing short of revolutionary.

## Funding

This paper was funded research deputy of 10.13039/501100005317Kermanshah University of Medical Sciences, Kermanshah, Iran.

## Declaration of competing interest

The authors declare that have no competing interests.
